# Mutational signature SBS8 predominantly arises due to late replication errors in cancer

**DOI:** 10.1038/s42003-020-01119-5

**Published:** 2020-08-03

**Authors:** Vinod Kumar Singh, Arnav Rastogi, Xiaoju Hu, Yaqun Wang, Subhajyoti De

**Affiliations:** Rutgers Cancer Institute, Rutgers the State University of New Jersey, New Brunswick, NJ 08901 USA

**Keywords:** Genome informatics, Cancer genomics

## Abstract

Although a majority of somatic mutations in cancer are passengers, their mutational signatures provide mechanistic insights into mutagenesis and DNA repair processes. Mutational signature SBS8 is common in most cancers, but its etiology is debated. Incorporating genomic, epigenomic, and cellular process features for multiple cell-types we develop genome-wide composite epigenomic context-maps relevant for mutagenesis and DNA repair. Analyzing somatic mutation data from multiple cancer types in their epigenomic contexts, we show that SBS8 preferentially occurs in gene-poor, lamina-proximal, late replicating heterochromatin domains. While SBS8 is uncommon among mutations in non-malignant tissues, in tumor genomes its proportions increase with replication timing and speed, and checkpoint defects further promote this signature - suggesting that SBS8 probably arises due to uncorrected late replication errors during cancer progression. Our observations offer a potential reconciliation among different perspectives in the debate about the etiology of SBS8 and its relationship with other mutational signatures.

## Abstract (web summary)

Vinod Kumar Singh, Arnav Rastogi, et al. study etiology of mutational signature 8 (SBS8) in human cancers. They develop genome-wide composite epigenomic context maps related to mutagenesis and DNA repair. They report that SBS8 preferentially occurs in heterochromatin and likely arises from late replication errors during cancer progression.

## Introduction

**Fig. 1:**
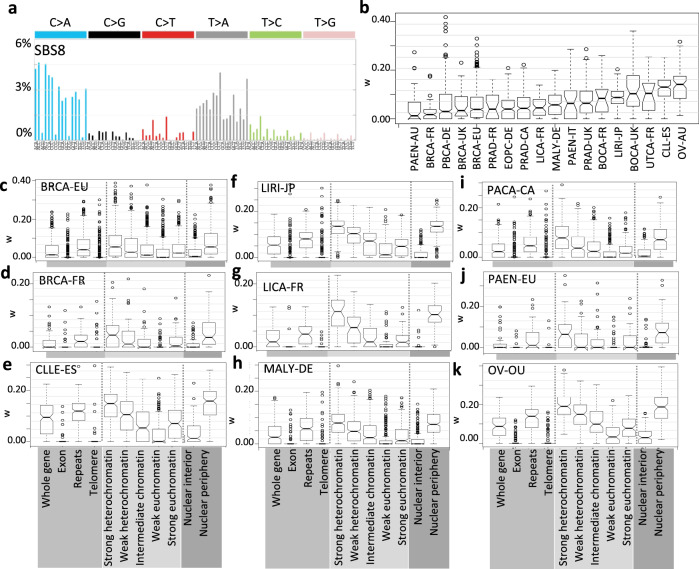
Genomic and epigenomic context-preference for SBS8 in different cancer cohorts. **a** Relative frequencies of single base substitutions at 96 trinucleotide contexts for mutational signature SBS8. **b** Boxplots showing distributions of weights of SBS8 (w) in multiple cancer cohorts. **c**–**k** Boxplots showing distributions of weights of SBS8 (w) in different genomic, epigenomic, and nuclear localization contexts in multiple cancer types listed above. Acronyms of the cancer cohorts are listed at the top left corner. See Supplementary Data 1 for description of the cancer cohorts including the number of samples.

During development and aging, DNA damage and repair defects result in accumulation of somatic genomic alterations, including point mutations, genomic rearrangements, and ploidy changes that contribute to aging, cancer initiation and progression^1,2^. Even though a majority of the somatic mutations are not disease-drivers, mutational signatures, i.e., their patterns of genetic changes provide insights into past exposure to mutagens, mechanism of DNA damage, DNA repair defects, and extent of genomic instability^2–7^. Nonnegative matrix factorization-guided deconvolution of somatic mutations in their sequence contexts across all major cancer types has identified a number of mutational signatures^8,9^. Mutational signature 8 (SBS8; Fig. 1a) is widely present in multiple cancer types^8^, but its etiology is not well understood. SBS8 has a broad trinucleotide context preference, although C > A, C > T and T > A substitutions are proportionally over-represented. It is not associated with any known exogenous mutagen exposure, and does not show major transcriptional strand bias. Emerging reports suggested that SBS8 might be associated with genomic instability, perhaps concurrently with SBS3—signature of deficiency of double strand break repair via homologous recombination (HRD)^10^ and that nucleotide-excision repair deficient tumors have elevated burden of SBS8^11^, but SBS8 is also detected in tumors with no overt NER pathway defects or HRD-related genomic instability^8,9,12^. Therefore, mechanistic basis of SBS8 is still debated.

Here, we develop an epigenomic composite context-map of the genome incorporating genomic, epigenomic, and cellular process features that are relevant for mutagenesis and DNA repair, and examine whether SBS8 preferentially occur in specific epigenomic context that could provide etiological insights. We also assess potential crosstalk between SBS8 and selected other signatures within and across epigenomic contexts. Our context-guided analysis provides a rational roadmap for investigating etiologies of the emerging mutational signatures.

## Results

### SBS8 is depleted in exons and enriched in heterochromatin

We analyzed mutational signatures associated with somatic point mutations identified from whole-genome sequencing data for 18 cancer cohorts from the International Cancer Genome Consortium (ICGC)^13^ (Fig. 1b; Supplementary Data 1); the selected cancer types have diverse tissue-of-origin, and different exposures to endo- and exogenous mutagenic processes, which allow us to decouple tissue-dependent and context-dependent effects. SBS8 was present with sufficient footprints in most of the cohorts.

Since the mechanisms of endo- and exogenous DNA damage and repair preferences depend on local sequence, chromatin, and nuclear contexts^14^, we segmented the genome based on (i) genomic contexts: exons, whole genes (including exons and introns), repeats, and telomere, (ii) epigenomic contexts: strong heterochromatin, weak heterochromatin, intermediate chromatin, weak euchromatin, and strong euchromatin, and (iii) nuclear localization contexts: lamina-proximal regions in the nuclear periphery and inter-lamina regions in the nuclear interior—and estimated the proportions of different mutational signatures including SBS8 within and across various genomic and epigenomic contexts. Although not exhaustive, these contexts are associated with major classes of mutagenesis processes, overall genome maintenance, and DNA repair pathway choices^2,8,15–17^—which can aid to per exclusionem, i.e., exclude unlikely possibilities while generating testable hypotheses about plausible etiology of mutational signature of interest and guide downstream analyses.

Among different genomic contexts, SBS8 was depleted in the telomere and exonic regions in nearly all cancer types analyzed (Fig. 1c–k). At the level of whole genes, presence of SBS8 was detected, but that contribution primarily came from the intronic regions. We observed consistent results in most cancer types, including those that were represented by multiple independent cohorts. It was relatively over-represented in repeats compared to exons (Wilcoxon rank sum test; combined *p* value across all cancer types <1e−05), although the SBS8 mutational signature did not indicate any specific preference for homopolymeric tracks or specific repeat motifs (Fig. 1a).

We next focused on chromatin and nuclear localization contexts, which unlike the genomic contexts, are tissue dependent. Since the cell of origin is not known for many cancer types and/or relevant tissue-specific epigenetic data is available for limited tissue types, we first used tissue-invariant chromatin and nuclear localization data^18^ for the initial epigenomic analysis. Subsequently we also repeated the analyses using tissue-specific data for selected cancer types and obtained consistent results, as discussed later. In all cancer types analyzed, the SBS8 was significantly more enriched in heterochromatin than euchromatin regions (Fig. 1c–k; Spearman correlation; combined *p* value across all cancer types <1e−05). Similarly, it was significantly more over-represented in lamina-proximal regions in the nuclear periphery than inter-lamina regions in the nuclear interior (Fig. 1c–k; Wilcoxon rank sum test; combined *p* value across all cancer types <1e−05). We did not observe any specific enrichment for SBS8 in fragile sites (Supplementary Fig. 1). The results were consistent across the cancer cohorts, including those representing similar cancer types.

We considered the possibility that the number of mutations attributed to a mutational signature (signature weight × number of mutations/Mb) could be actually higher in a given context, even when there is an apparent decrease in relative proportion of that signature due to an excess of other signatures. We found no evidence supporting that possibility confounding our conclusions about the observed difference in preference of SBS8 for heterochromatin over euchromatin. In fact, somatic mutation rate in gene rich euchromatin is lower than that in the heterochromatin regions^18–20^. Taken together, SBS8 is depleted in exonic regions, euchromatin, and nuclear interior, and proportionally more common in repeat regions, heterochromatin and nuclear periphery.

### Composite epigenomic context preference of SBS8

**Fig. 2:**
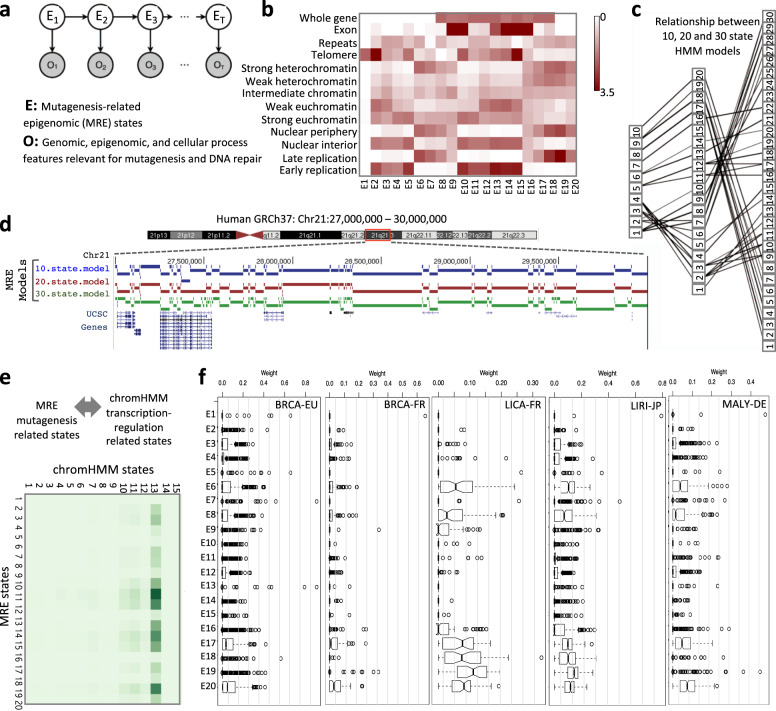
Composite epigenomic context analysis of SBS8. **a** A schematic representation of the Hidden Markov Model used to identify mutagenesis-related epigenomic (MRE) states integrating genomic, epigenomic, and cellular process features relevant for mutagenesis and DNA repair. **b** Enrichment score of the features for the MRE states in a 20-state model. Descriptions of the MRE states are provided in Supplementary Data 1. Enrichment values of exon for E9–10 and 14–16 are >9. Contrast is saturated for values >3.5. **c** Relationship between MRE states from the 10, 20, and 30 state models. **d** Annotation of chromosome 21:27–30 Mb regions using 10, 20, and 30 state models and UCSC Genes are shown in breast epithelial cell type. Genomic coordinates of the MRE annotations from the 20-state model are provided in Supplementary Fig. 2 and Supplementary Data 3. **e** Relationship between MRE states and ChromHMM chromatin states. **f** Boxplot showing distributions of weight of SBS8 in different MRE contexts for multiple cancer types. See Supplementary Data 1 for description of the cancer cohorts including the number of samples.

The nucleotide, genomic, and epigenomic features are not independent, and combinatorically influence DNA damage and repair^14^. Therefore, feature-by-feature analysis may be inadequate to appreciate complex patterns of context-dependent mutagenic processes. At this end, we developed an epigenomic composite context-map of the genome using a Hidden Markov Model, incorporating genomic features as well as tissue-dependent epigenomic and cellular process features that are relevant for mutagenesis and DNA repair (Fig. 2a). The HMM approach allowed us to describe combinatorial patterns of relevant epigenomic features using a small number of composite contexts that are prevalent in the genome, and flexibly determine the resolution of the context-map by adjusting the number of such contexts. This offered a distinct advantage over considering exhaustive combinations of features, because the number of possible combinations increases exponentially with an increase in the number of features considered, and some combinatorial contexts are rarely observed in mammalian genomes.

We jointly annotated mutagenesis-related epigenomic (MRE) states for multiple cell types from the ENCODE project^21^ (see “Methods” for details) and for downstream analyses used a 20-state model which was computationally robust and biologically interpretable. For instance, E6 and E16–20 contexts are marked by late replicating heterochromatin, but they differ in terms of presence of exonic, intronic, intergenic, and repeat contexts (Fig. 2b). Likewise, E9–10 and E14–16 are exonic regions, but differ in terms of their chromatin, nuclear localization, telomere, and replication contexts. Joint annotation of MRE states across cell types meant that the interpretation of the MRE state is invariant across cell types, but genomic segments attributed to that state might differ between cell types, primarily due to difference in cell-type dependent epigenomic makeups. A predominantly parent–child relationship between the MRE states in the lower and higher order models was observed, such that the MRE states are mostly subclassified into finer sub-states in corresponding higher order models (Fig. 2c), which would allow us to control resolution of the context-map by selecting appropriate state model if necessary. For instance, a single state (_10_E9) in the 10-state model was subdivided into E18 and E19 in the 20-state model. Interpretation of the contexts and their genome-wide prevalence in different cell types are provided in Supplementary Data 2 and 3 respectively, while an example of MRE annotations from the 10, 20, and 30 state models for chromosome 21 in breast epithelial cell type are shown in Fig. 2d and Supplementary Fig. 2. Our approach is conceptually similar to that adopted to identify chromHMM states^22^, which are specific for transcriptional regulation. But unlike chromHMM we incorporated genomic and epigenomic features that are specifically relevant for replication, DNA damage and repair, such that (i) the composition of chromHMM and MRE states are different, and (ii) genome-wide distributions of MRE states are fundamentally different (Fig. 2e). The composite MRE states are more broadly distributed genome-wide than the chromHMM states which show variations primarily around coding and regulatory regions which cover only about 2–5% of the genome.

SBS8 was over-represented in MRE state E20 (Fig. 2f), which is late replicating heterochromatin across multiple cancer types, but also in E6 and E17 states, which showed similar contextual composition. In liver cancer, SBS8 was also common in E18 and E19 contexts (Fig. 2f), which shared the late replication patterns. Although there were minor variations between the cancer types, SBS8 was prominently present in late replicating heterochromatin and depleted in early replicating euchromatin in all cancer types analyzed. Based on the feature-by-feature and composite context analyses, we conclude that SBS8 is prevalent in late replicating, repeat-rich, heterochromatic regions over early replicating, gene-rich, euchromatic regions, as consistently observed in tissue-invariant feature-by-feature and tissue-specific composite context analyses in all cancer types.

### Inference of etiology of SBS8 per exclusionem

SBS8 was present in multiple cancer cohorts, including those not attributed to environmental exposure and its nucleotide substitution pattern did not overlap with any known exogenous mutagen. This suggests that it is unlikely to occur due to external agents, and might arise via endogenous processes. The context-guided analysis further indicates that SBS8 rarely occurs in certain epigenomic contexts, allowing us to exclude certain classes of mutagenic processes from consideration. Unlike other mutational signatures (e.g., SBS4, SBS12, SBS16, and SBS19) that are specifically associated with transcription-coupled DNA damage and repair, SBS8 was depleted in exons (Fig. 1c–k) and did not have strong transcriptional strand bias^23^. In addition, lack of enrichment of SBS8 in euchromatin regions and lack of motif-preference, as reflected in the broad trinucleotide context-preference of SBS8, provide no support for transcription factor-mediated or motif-specific mutagenesis. Recent reports suggest that SBS8 might be associated with HRD signature SBS3^10^ and that nucleotide-excision repair deficient tumors have excess of SBS8^11^. But SBS8 is present in tumors with no obvious NER pathway defects or homology-mediated repair defects^9^—suggesting that there are additional mutagenic mechanisms involved. On the other hand, prevalence of SBS8 showed the most systematic difference with chromatin and nuclear localization across all cell types. In the composite context analysis chromatin and replication timing were the key factors distinguishing the enriched states such as E6, E17–20 from others. We know that (i) chromatin is the primary determinant of replication profile of eukaryotic genomes^24^, and (ii) a vast majority of somatic mutations arise as a result of replication errors, and late replication is particularly error-prone^2,18,20^—motivating us to investigate plausible roles of replication in the etiology SBS8.

### Replication context preference of SBS8

**Fig. 3:**
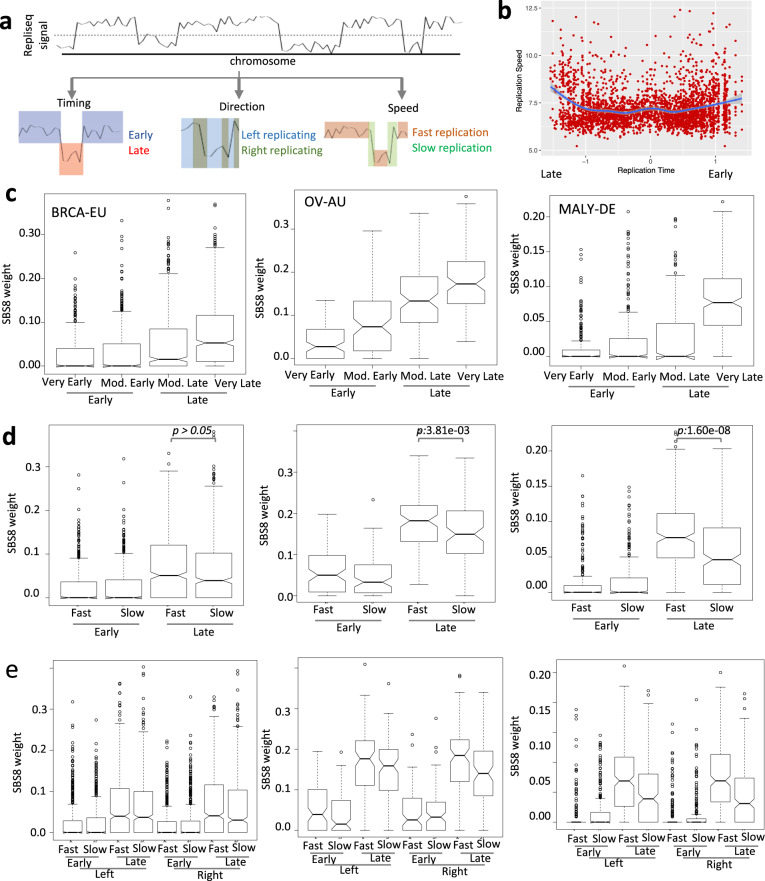
Replication context analysis of SBS8. **a** Schematic representation of inference of replication timing, direction of fork progression, and replication speed from repliseq data. **b** Scatterplot showing changes in replication speed with replication timing in MCF7 breast cancer cell line, which shows an increase in replication speed late during replication. Similar results are observed for other cell lines. **c** Boxplot showing distributions of weight of Signature 8 in replication timing contexts in breast cancer (BRCA-EU), ovarian cancer (OV-AU), and lymphoma (MALY-DE). **d** Boxplot showing distributions of weight of Signature 8 in combinations of replication timing and speed contexts in breast cancer (BRCA-EU), ovarian cancer (OV-AU), and lymphoma (MALY-DE). *p* Values for comparisons between fast and slow replication speed in late replication contexts are listed; combined *p* value for the three cohorts using Fishers method is 3.45e−09. **e** Boxplot showing distributions of weight of Signature 8 in combinations of replication timing, speed, and direction contexts in breast cancer (BRCA-EU), ovarian cancer (OV-AU), and lymphoma (MALY-DE). See Supplementary Fig. 3 for similar results for other cancer cohorts. *p* Values for comparisons between left and right replication direction were not significant, when analyzed in the context of combinations of replication timing and speed in the cohorts. See Supplementary Data 1 for description of the cancer cohorts including the number of samples.

Using Repli-seq data^25^ from multiple human cell and tissue types, we annotated genomic regions as early or late replicating in respective cell types, and further inferred both replication direction relative to the reference strand in the genome and speed of replication from the pattern of transition of replication timing along the genome (Fig. 3a). Although replication speed showed regional variations, in general, it increased towards very late replication in all cell types analyzed (Fig. 3b, Supplementary Fig. 3). This is in agreement with reports that late replication is marked by low origin density but higher replication speed (1.5–2.3 kb/min) than that of early replication domains (1.1–1.2 kb/min)^26^.

Analyzing the proportion of SBS8-associated somatic mutations in tumor genomes in the replication contexts from closely related cell types, we found that late replicating regions had significant excess of SBS8 compared to early replicating regions in cancer (Fig. 3c, Supplementary Fig. 3; Wilcoxon rank sum test; combined *p* value <1e−05), and within late replication timing contexts, high replication speed was associated with increased burden of SBS8 (Fig. 3d**;** Wilcoxon rank sum test; combined *p* value <1e−05). We also found similar results using tissue-invariant replication timing data on all cancer cohorts (Supplementary Data 4), and our findings are consistent with the observations in breast cancer^27^. We did not find significant difference between left and right replicating strands in terms of the burden of SBS8 (Fig. 3e), that is in agreement with previous reports that SBS8 displays no major replication strand bias^28^. There were no evidence that SBS8 was preferentially enriched for kataegis or extended processivity^27^, i.e., sets of consecutive mutations with same reference allele attributed to the same signature. Common and early replicating fragile sites were not enriched for SBS8 (Supplementary Fig. 1), especially relative to late replicating regions in general—indicating that replication fork collapse may not be a major source of SBS8.

Anyhow, Fig. 3d suggests that both replication speed and timing likely have independent effects, although replication timing might have proportionally higher effect size. It is known that average replication fork speed increases markedly in presence of A + T)^29^, and that dATP/dTTP proportions increase during late replication (Supplementary Fig. 4) and drive mutation spectrum that favors AT nucleotides at late S-phase^30^, and indeed SBS8 has a preference for substitution to A or T. Moreover, although replication errors occur throughout S phase, early replication errors are more effectively repaired by mismatch repair and nucleotide excision repair (NER) than those replicated late^31,32^, which also contribute to increased mutation burden in late replicating regions^20^.

### SBS8 and genome maintenance

Uncorrected replication errors have potentials to stall replication, trigger checkpoint activation, and promote genomic instability^32^. *ATR* mediated DNA damage sensing for single strand breaks and *CHEK1/2*-mediated checkpoint activation are tightly coupled such that mis-incorporated bases trigger DNA damage sensing and checkpoint activation. Checkpoint defects are common in cancer genomes, which might allow the cells to proceed through the cell cycle without appropriate repair of these lesions resulting in mutations. Therefore, if SBS8 is indeed due to replication errors, we should detect additional evidence at genomic and cell cycle contexts. At this end, we grouped the tumors in respective cohorts into three groups based on purity adjusted *ATR* expression—low (0–33%), middle (33–67%), and high (top 66–100%), and found that the *ATR*-high tumors indeed have high proportion of SBS8 in somatic mutations accumulated in late replicating domains (Supplementary Fig. 5); in contrast, when the tumors are grouped according to purity adjusted *CHEK1* or *CHEK2* expression, low checkpoint gene expression was associated with high proportion of SBS8 in late replicating domains (Supplementary Fig. 5). In fact, the tumors with high *ATR* and also low *CHEK1* or *CHEK2* expression had proportionally more SBS8 compared to other combinations (Supplementary Fig. 6). These observations are consistent with a model that checkpoint defects are associated with high prevalence of SBS8.

We note that tumor transcriptome changes with time such that current expression of those genes is a poor proxy of their past expression, and it is not possible to obtain expression data from the time-point when dividing cells accumulated the observed somatic mutations in the genomes. Moreover, components of DNA repair pathways are regulated at transcriptional and post-translational levels, such that correlative data need to be interpreted keeping the caveats in the mind. Thus, next we analyzed data on acquired mutations in clonally derived cell lines with checkpoint defects, i.e., that had no functional copy of multiple DNA repair pathway genes including *CHEK2*^33^. The catalog of acquired mutations in the *CHEK2*−/− clones had predominantly background genome maintenance signature (dubbed BG signature) while contribution from homology repair defect signature (SBS3 like) was minimal^33^. We observed that the BG signature had one of the highest cosine similarity with SBS8 (0.663). Taken together, these observations suggest that the checkpoint defects generate a mutational signature that bear high similarity with SBS8.

### Crosstalk between SBS8 and other mutational signatures

**Fig. 4:**
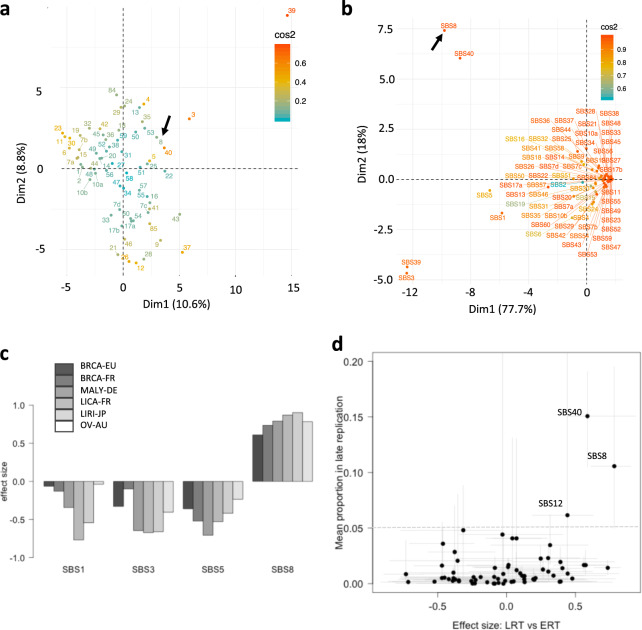
Crosstalk between SBS8 and other mutational signatures. **a** PCA plot showing different mutational signatures projected based on their trinucleotide frequencies. Cosine similarity is shown to the right. **b** PCA plot showing different mutational signatures projected based on their weights in different epigenomic contexts. SBS8 is marked with an arrow. Cosine similarity is shown to the right. **c** Effect size of selected mutational signatures SBS1, SBS3, SBS5, and SBS8 in late replication contexts relative to early replication contexts in different cohorts. Negative values indicate preferential occurrence in early replication contexts. **d** Scatterplot showing mean proportion of each signature in late replication against its effect size between early and late replication contexts. SBS8, SBS40, and SBS12 are marked. Whiskers indicate the maximum and minimum values across the cancer cohorts. See Supplementary Data 1 for description of the cancer cohorts including the number of samples.

We investigated association between SBS8 and other mutational signatures within and across genomic and epigenomic contexts to understand context-dependent interplay between these signatures for a number of reasons. First, mutagenesis and DNA repair do not occur in isolation and there is crosstalk between different mutagenic, DNA damage sensing, and repair pathways^14,16,32^. Second, there are ongoing debates about computational deconvolution of mutational signatures, especially those with broad-spectrum substitution patterns (e.g., SBS3, SBS5, and SBS8). Third, multiple signatures might represent variations of the same underlying process in a context-dependent manner^14^. At this end, we first projected the signatures in a PCA plot based on their trinucleotide contexts. In terms of trinucleotide context frequencies, SBS8 has some similarities with other mutational signatures such as SBS3 and SBS5 (Fig. 4a), which also have broad-spectrum nucleotide substitution patterns and are often discussed together.

Nonetheless, when the epigenomic and replication context preferences were analyzed, differences among the signatures became evident. We used PCA plot to compare epigenomic context-based proportions of different signatures (Fig. 4b) based on genomic, chromatin, and nuclear localization features from Fig. 1; SBS8 showed similarity with SBS40 and was distinct from other broad-spectrum nucleotide substitution signatures such as SBS1, SBS3, and SBS5. Like SBS8, SBS40 is also a broad-spectrum substitution-based signature with unknown etiology. Among the closely related broad-spectrum substitution-based signatures, only SBS8 shows consistent and significant preference for late replication, while SBS1, SBS3, and SBS5 consistently were depleted in late replication context, in all cancer cohorts including those representing similar cancer types (Fig. 4c**;** Supplementary Fig. 7). We also observed similar results using cell type dependent replication timing data. In a pan-signature analysis, among the signatures with sufficient presence (>5% proportion) in the cohorts, SBS8 showed the highest effect size in to discriminate early and late replication contexts (Fig. 4d). Apart from the SBS8, only SBS40 and to some extent SBS12 had high proportional contribution among somatic mutations in late replicating regions in all cancer types, and also high effect size to discriminate early and late replication contexts (Fig. 4d**;** Supplementary Fig. 8). SBS12 is a NER signature marked by excess of T > C, which is distinctly different from SBS8, but the etiology of SBS40 is unknown. Based on their similarity both at the trinucleotide level and presence in different epigenomic and replication contexts, we argue that SBS8 and SBS40 might be related.

Next, we investigated whether SBS8 in late replication context correlated with any other mutational signature, especially those known to be genome maintenance-related, in early replication context complementing it. Association of SBS8 with other mutational signatures was cancer type dependent and context-dependent (Supplementary Data 3). The proportion of different NER signatures (e.g., SBS7, SBS19, and SBS32) in early replicating regions correlated with the proportion of SBS8 in late replication in multiple cohorts. But no single signature, NER-related or otherwise, correlated with SBS8 within and across epigenomic contexts in a majority of cancer types tested. In liver cancer the proportion of SBS8 in late replication context correlated with SBS5 in early replication, while in breast and ovarian cancer, proportion of SBS8 in late replication significantly correlated with the proportions of Signature SBS3 and SBS1 in early replicating contexts. Our observations are consistent with reports that tumors with *BRCA1*/*BRCA2* deficiency have high burden of SBS8^10^, but indicate that such associations are tissue specific.

Replication errors have potentials to cause DNA double strand breaks, rearrangements, and genomic instability, and the burden of genomic structural variations in cancer genomes is known to be high in late replicating heterochromatin domains^34,35^. Integrating somatic structural variation data for the ICGC cohorts we observed that, in selected cancer types such as breast and ovarian cancer, the proportion of SBS8 genome-wide strongly correlated with increased frequency of somatic structural variations in breast and ovarian cancers (Supplementary Fig. 9); we observed similar results based on proportion of SBS8 in late replicating regions in these cohorts. Our observations are consistent with that based on SBS3 published reports in breast cancer^10^ and suggest that association between SBS8 and genomic structural instability might be cancer type dependent. It is possible that repair pathway defects augment both late replication errors and genomic instability, which might drive the observed associations.

### SBS8 is uncommon in nonmalignant tissues

Analyzing de novo germ line mutations from whole genome sequencing of 250 parent–offspring families^36^, we found that proportion of SBS8 signature overall was very low in the germ line, and there was no significant difference between its weight in late (mean: 0.0142) and early replicating regions (mean: 0.0114; *p* value > 0.05; Supplementary Fig. 10). This was not due to modest mutation count per sample; we observed similar results when the analysis was performed at the cohort-level after mutations from all samples were pooled. Likewise, mutational signature analysis of nonmalignant somatic did not show any substantial contribution of SBS8 at a genome-wide level^37–40^, which contrasts the patterns observed in cancer genomes in all major cancer cohorts. In addition, unlike SBS1 and SBS5, it does not show any prominent clock-like properties, i.e., age-associated increase in burden of associated mutations in somatic genomes^41^. On the other hand, when the tumors were grouped based on stage, the weight of SBS8 in late replicating regions increased with pathological staging in multiple cancers (Supplementary Fig. 10). Therefore, SBS8 mutational signature appears to be rare in nonmalignant cells, but likely arises during cancer progression.

## Discussion

**Fig. 5:**
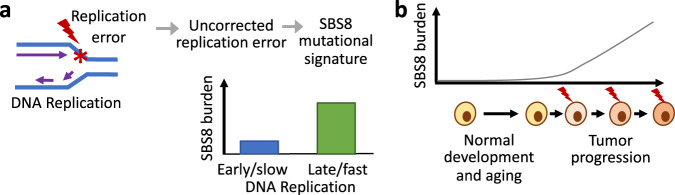
Schematic representation showing emerging characteristics of SBS8 mutational signature. **a** SBS8 is suspected to arise due to replication errors, and has higher burden in context of late and fast DNA replication. **b** SBS8 is relatively uncommon in de novo germ line mutations and somatic mutations in nonmalignant tissues, but has progressively increased burden in advanced tumors, which have sustained growth signal, impaired genome maintenance, and/or checkpoint defects.

Our results indicate that SBS8 preferentially occur in late replicating gene poor, lamina-proximal heterochromatin regions, where replication timing and speed emerge as major determinants (Fig. 5a). In contrast, replication strand bias appears to have no major effects on SBS8. Our results are consistent with that reported in breast cancer^27^. It is possible that high replication speed increases replication stress and elevates base-line error-rates, as observed in viral genomes^42^. Imbalance in the nucleotide pools may further promote C > A:G > T and T > A:A:T substitutions during late replication. While early S phase templates have more time to recognize and repair mutations prior to mitosis, late replication errors may persist, especially in tumor genomes where strong growth signal and/or checkpoint defects drive the cell cycle to progress to mitosis without sufficient replication-coupled or post-replicative repair. It is known that mismatch repair and NER manage to correct replication errors more effectively during early replication^31,32^, and when either the nucleotide excision or mismatch repair pathway is defective, mutations are relatively more evenly distributed throughout the genome^31^. Indeed, SBS8 is rare in the germ line mutations and somatic mutations in nonmalignant tissues, while tumor genomes, particularly those with checkpoint defects, DNA repair defects, or signatures of genomic instability have high prevalence of SBS8 (Fig. 5b, Supplementary Fig. 10)—which is in line with the facts that uncorrected replication errors trigger checkpoint activation, and promote somatic mutations and overall genomic instability^34,43^. Therefore, SBS8 might be a marker of tumorigenesis, although further work is needed to firmly establish that.

Our observations offer a potential reconciliation among different perspectives in the debate about the etiology of SBS8. It was recently suggested that NER pathway deficiency, particularly in CC»AA context contribute to SBS8 burden^11^ while others showed that *BRCA1* and *BRCA2* mutant tumors have high burden of SBS8 associating it with HRD and genomic instability^10^. Our findings suggest that such associations are not necessarily mutually exclusive, and might be context-specific especially since SBS8 is also detected in tumors with no overt HRD or NER defects. It is possible that inefficient global genomic NER during late replication contributes to this signature, and uncorrected lesions could lead to both point mutations and genomic instability, particularly in HRD contexts. Furthermore, replication and genome maintenance are complex processes, involving interaction among multiple repair-related pathways^14,16,32^ such that associated mutations arising may not be explained by a single mutational signature; rather owing to differences in cell-type dependent differences in cell cycle, endogenous mutagenesis, chromatin remodeling, and repair processes there may be tissue-dependent crosstalk between multiple mutational signatures.

Context-guided analysis can provide crucial insights into mutagenic processes operating on the genome. Our approach is complementary and synergistic to the attribution method^27^ given that the former can identify prevalence of different mutational signatures in a given epigenomic context, while the latter can probabilistically assign individual mutations therein to most likely mutational signatures, providing etiological insights at different genomic resolutions. At this end, our context-guided approach is applicable not just to the SBS8, but provides a rational roadmap for investigating underlying mechanisms of the emerging mutational signatures associated with single- and dinucleotide substitutions, InDels, and rearrangements.

## Methods

### Cancer cohort datasets

We analyzed somatic point mutation data for multiple cancer types from the International Cancer Genome Consortium (ICGC release 28)^13^. The cancer types selected had diverse tissue-of-origin, mutation burden, and oncogenic drivers, which enabled us to draw etiological inference about the mutational signatures without any tissue-dependent bias. After removing samples with <500 somatic point mutations from whole-genome sequencing, we had 20–569 samples (median: 145) per cohort for downstream analyses. A summary of the cohorts included in the study are listed in Fig. 1b and Supplementary Data 1. In some cohorts, a subset of the samples had structural variation and/or RNAseq-based expression data available. We also obtained data on 11,020 de novo germ line mutations identified using whole genome sequencing of 250 Dutch parent–offspring families from the Netherland Genome Project^36^, which profiled 231 trios, 11 quartets with monozygotic twins, and 8 quartets with dizygotic twins from 11 of the 12 Dutch provinces without ascertaining on the basis of phenotype or disease.

### Mutational signature analysis

We obtained consensus single base substitution (SBS) mutational signatures (version 3) from the COSMIC database^23^ that included 49 SBS, 11 doublet base substitution, and 17 indel signatures. These signatures were identified by Alexandrov et al.^9^ who used nonnegative matrix factorization techniques to analyze nucleotide contexts of somatic mutations in tumor genomes from the ICGC cancer cohorts^13^. SBS8 (Fig. 1a) is one of the single nucleotide substitution signatures, which has remained broadly consistent with that reported in the previous versions (e.g., SBS mutational signatures from the COSMIC database version 2). It can be challenging to directly apply the attribution method^27^, i.e., compute probability for individual mutations to be caused by a given mutational signature in a given sample, especially for mutational signatures with broad trinucleotide context preferences (e.g., SBS8). Therefore, we adopted a complementary approach, and segmented the genome in different genomic/epigenomic contexts, identified the somatic mutations in such contexts in each sample, and estimated the proportion of mutational signatures based on those somatic mutations. We computed the proportions of the signatures in the cohorts using deconstructSigs^44^, and took into consideration the discussion about best practice guidelines for mutational signature extraction^45–48^. This allowed us to directly compare contributions of different mutational signatures across genomic contexts, and across patients such that our inferences are unaffected by length of different genomic contexts or difference in overall mutation burden between tumors. We also computed the burden of mutational signatures (signature weight × number of somatic mutations/Mb in that context) genome-wide or in specific genomic context. Our key conclusions were unchanged when we compared mutational signature burden, or used legacy SBS mutational signature COSMIC version 2, which had 30 signatures.

### Genomic and epigenomic contexts

The mechanisms of DNA damage and repair depend on local sequence, chromatin, and nuclear contexts^14^. Thus, we defined (i) genomic contexts: exons, whole genes (including exons and introns), repeats, and telomere, (ii) epigenomic contexts: strong heterochromatin, weak heterochromatin, intermediate chromatin, weak euchromatin, and strong euchromatin, and (iii) nuclear localization contexts: lamina-proximal regions in the nuclear periphery and inter-lamina regions in the nuclear interior, and compared presence of different mutational signatures within and across contexts.

Some features (e.g., genomic contexts) are tissue-independent; in other cases, cell type specific data (e.g., replication timing) was used when available. Annotations for exons, genes, telomere, and repeat elements was obtained from the UCSC Genome Browser^49^. Tissue-dependent replication timing data were obtained from the Replication Domain database^50^. Tissue-dependent histone modification and chromatin data for selected human tissue types was obtained from the ENCODE project^21^. Tissue invariant early and late replication timing, giemsa-staining based chromatin, and lamina proximity data was obtained from Smith et al.^18^ and processed in a similar manner. Data on common fragile sites and early replicating fragile sites were obtained from published studies^35,51^.

We processed and analyzed somatic mutations in the contexts of genomic, epigenomic, chromatin, and nuclear features using mutSigTools R package (https://github.com/sjdlabgroup/MutSigTools). We computed the proportions of the mutational signatures in each sample in each context using deconstructSigs^44^. The proportion of SBS8 in tissue-invariant early and late replication contexts is provided in Supplementary Data 5. As a special case, we compared both proportion and mutation burden of mutational signatures between different contexts to assess whether the number of mutations attributed to a mutational signature (signature weight × number of mutations/Mb) is higher in a context, even when there is an apparent decrease in relative proportion of that signature due to an excess of other signatures. We found no evidence supporting that possibility confounding our conclusions about the observed difference in preference of SBS8 for heterochromatin over euchromatin, and late replication over early replication context. We used SigProfiler^9,52^ to extract de novo mutational signatures from somatic mutations in early and late replicating regions across all samples, and compare those with SBS8; but that did not resolve SBS8 into stable subsignatures with discrete and informative trinucleotide preferences.

### Composite context analysis

Trinucleotide, genomic, and epigenomic features are not independent, and synergistically impact DNA damage and repair^14^. We considered that biologically relevant combinations of such features could be represented by composite contexts, which could be modeled using a multivariate HMM (Fig. 2a). This approach enables probabilistic modeling of both the combinatorial presence/absence of multiple features and the spatial constraints of how these feature-combinations occur relative to each other across the genome. The former and latter are considered in the emission parameters and transition matrix of the multivariate HMM, respectively. We used Baum-Welch algorithm to learn the model parameters de novo in a data-driven manner on the basis of an unsupervised machine-learning technique that iteratively maximizes the model fit to the data. We jointly annotated MRE states for multiple cell types using ENCODE data for relevant cell lines (lung: IMR90; breast: MCF7; liver: HepG2; neuronal: SK–N–SH; hematopoietic lymphoid and myeloid cell types: GM12878 and K562, respectively)^21^. We computed enrichment scores for the features for classifying the MRE states (Fig. 2b), and generated MRE annotation for models with different number of states in different cell types. We further compared the MRE states with chromatin states identified by chromHMM^22^ that are relevant for transcription regulation. The features relevant and DNA damage and repair are distinct from those critical for chromHMM, although there are some overlaps. We computed the proportions of the mutational signatures in each sample in each composite context using deconstructSigs^44^ as before.

### Replication timing, strand bias, and directionality analysis

Replication is a highly coordinated cellular process; a majority of origins do not fire deterministically, rather origin firing occurs both individually and as clusters in the genome that correlate with local chromatin status. For tissue-dependent analysis, genome-wide replication timing data were fitted with cubic smoothing spline (smoothing parameter of 0.2) and analyzed as published^53,54^. Genomic regions with positive and negative value of replication timing scores were considered early and late replicating, respectively. From any origin of replication, the fork progress in both directions such that on one side, the genome reference strand is continuously replicated, while in the other direction it is replicated via Okazaki fragment. The sign of the replication gradient on the smoothed data provide information about direction of replication fork progression such that positive slope is represents left replicating strand and negative slope represents right replicating strand^53^. On both sides of the early replicating peaks the slope changes its polarity. Furthermore, we note that replication gradient along the length of the genome provides information about the speed of replication; when replication fork progresses fast, greater stretch of the genome is replicated between early and late S phases, compared to slow replicating segments where replication peaks and valleys are closely spaced. Thus, we labeled genomic regions as fast or slow replicating, if their absolute replication gradient is below or above median of the genome-wide values. Our key conclusions did not change after excluding the ENCODE back-listed genomic regions that are prone to technical artefacts (http://hgdownload.cse.ucsc.edu/goldenpath/hg19/encodeDCC/wgEncodeMapability/ wgEncodeDacMapabilityConsensusExcludable.bed.gz).

### Effect size calculation

For effect size estimation, we first calculated Wilcox signed-rank test statistic as $W = \mathop {\sum}\nolimits_{i = 1}^n {\left[ {sgn({{SBS}}_{i,{{LRT}}} - {{SBS}}_{i,{{ERT}}}).R_i} \right]}$, where ${{SBS}}_{i,{{LRT}}}$ and ${{SBS}}_{i,{{ERT}}}$ are proportion of i-th mutational signature in late and early replicating regions, respectively. Cell-type invariant replication timing data was used for effect size calculation for consistent processing of replication context in all cancer types. But we observed similar results using cell type specific data as well. To compute an effect size for the signed-rank test, the rank-biserial correlation was used. When *R* is the total rank sum, the effect size was computed as *W*/*R*^55^.

### Statistics and reproducibility

All statistical analyses were performed using R version 3.4.0. Sample sizes for respective cohorts are provided in Supplementary Data 1. Statistical tests and corresponding p values are listed for respective analyses. Correction for multiple testing using false-discovery rate was performed as appropriate. Combined *p* values were calculated using the Fisher’s method. In the boxplots, upper whisker is defined to be 1.5 × IQR more than the third quartile or the maximal value of the adjusted mutation rate (depending on which value is greater) and the lower whisker is defined to be 1.5 × IQR lower than the first quartile or the minimum value of the adjusted mutation rate (depending on which value is smaller) respectively, where IQR is the difference between the third quartile and the first quartile, i.e., the box length.

### Reporting summary

Further information on experimental design is available in the Nature Research Reporting Summary linked to this paper.

## Supplementary information

Supplementary Information

Supplementary Data 1

Supplementary Data 2

Supplementary Data 3

Supplementary Data 4

Supplementary Data 5

Reporting Summary

Description of Additional Supplementary Files

## Data Availability

This study used publicly available datasets. Composite epigenomic states are provided as Supplementary Data 3. Any other data are available from the corresponding author upon request.

## Code availability

The scripts used to process and analyze the mutation data are available at https://github.com/sjdlabgroup/MutSigTools, and/or can be requested from the corresponding author.
