# Supplementary material for: Mutational signature SBS8 predominantly arises due to late replication errors in cancer
Source: Commun Biol. 2020 Aug 3;3:421. doi: 10.1038/s42003-020-01119-5 (PMC7400754; doi:10.1038/s42003-020-01119-5)
Supplement: Supplementary file 1 — Supplementary Information [file 42003_2020_1119_MOESM1_ESM.pdf]

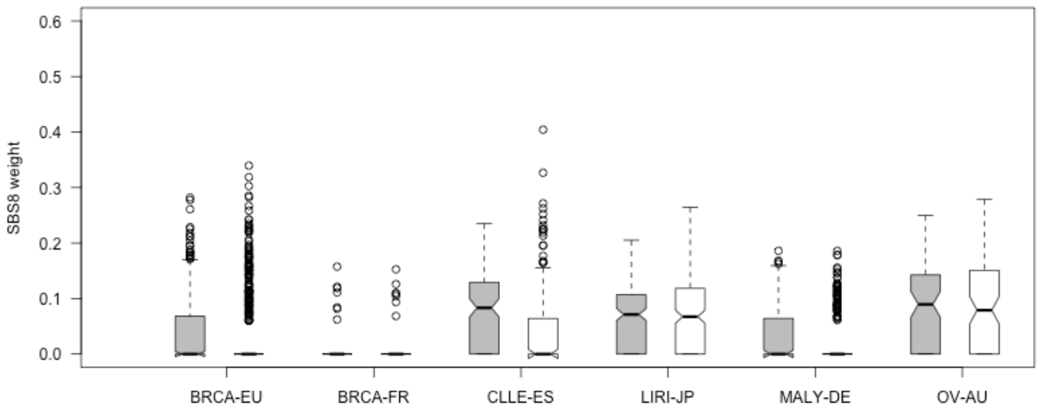

Supplementary Figure 1: Boxplots showing proportion of SBS8 weight in common fragile sites (CFS, light grey) and early replicating fragile sites (white) in multiple cancer types.

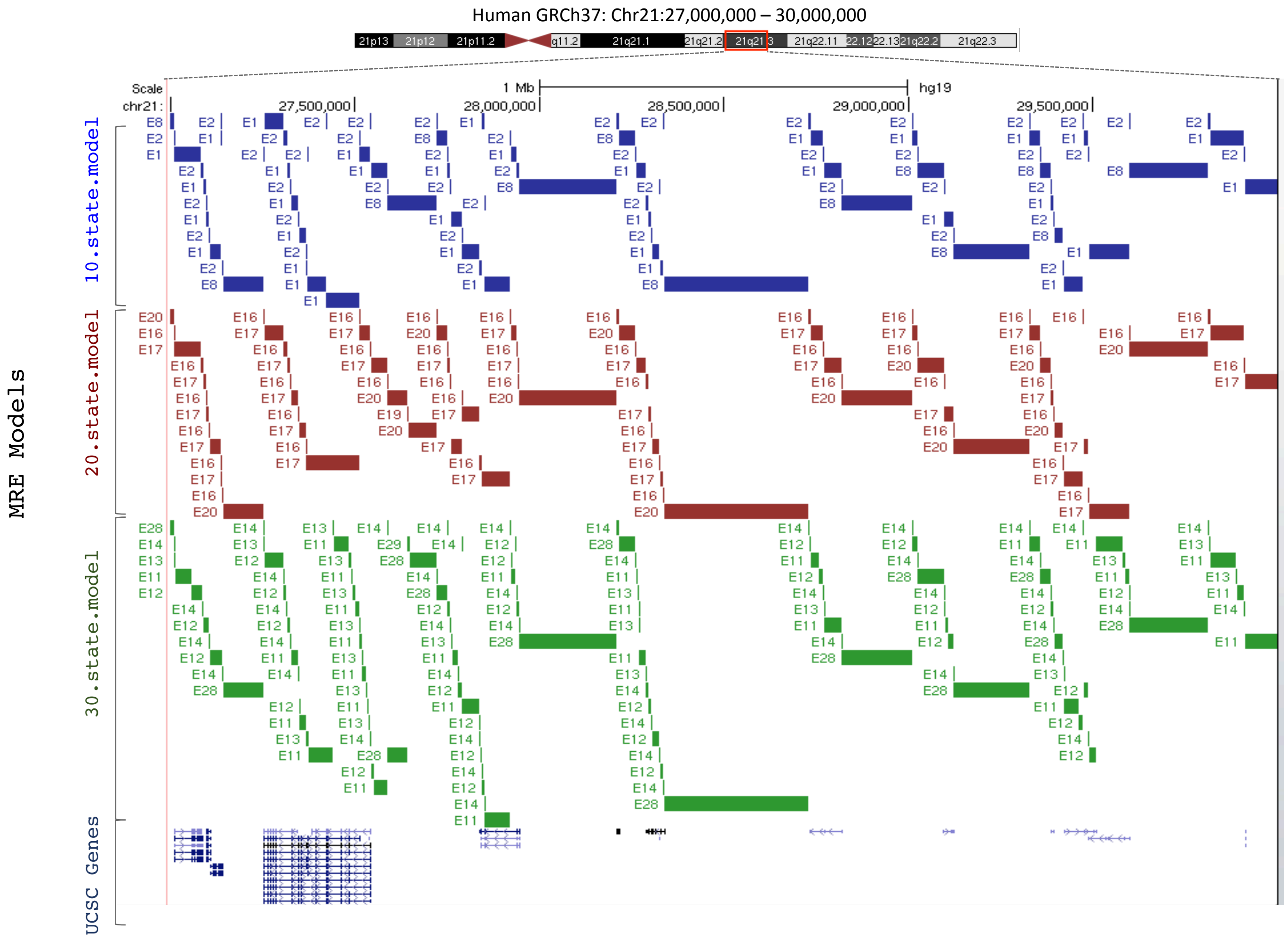

Supplementary Figure 2: Annotation of chromosome 21:27Mb - 30Mb regions using 10, 20, and 30 state models and UCSC Genes are shown in breast epithelial cell type.

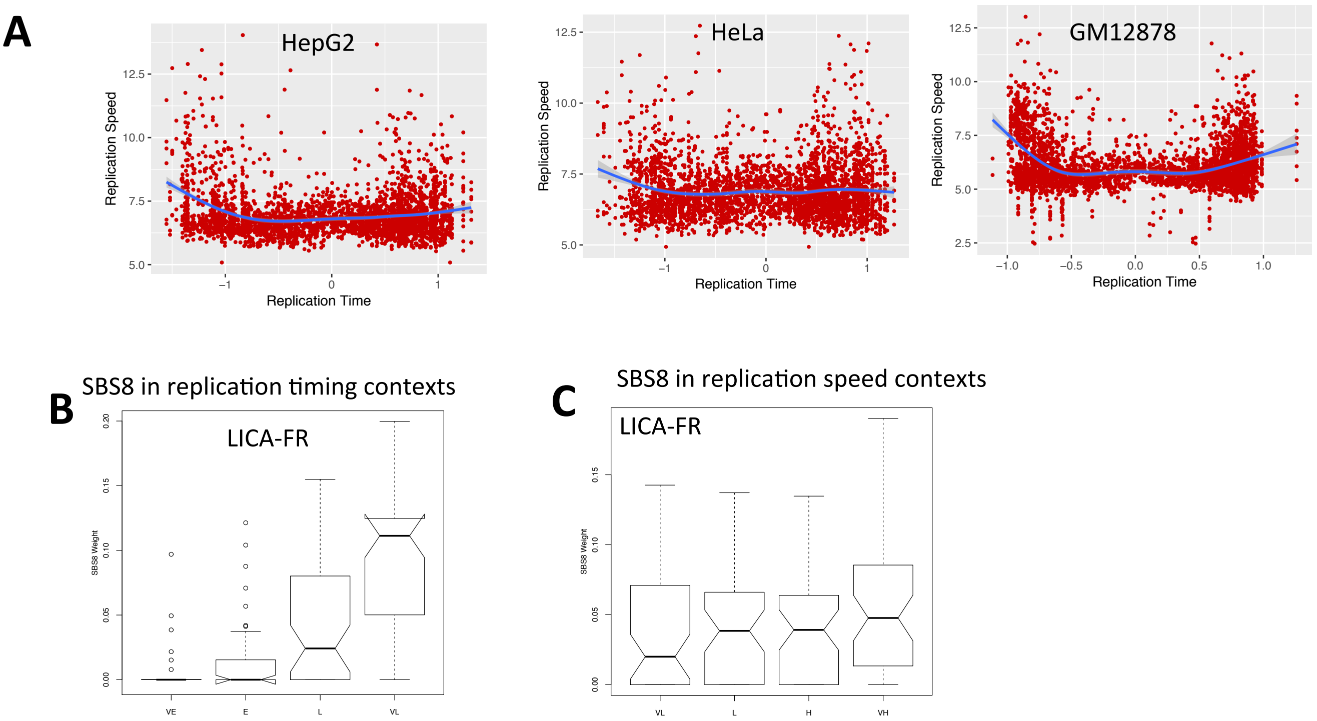

Supplementary Figure 3: A) Scatterplot showing changes in replication speed with replication timing in multiple cancer types. B) Boxplot showing distributions of weight of Signature SBS8 in very early (VE), early (E), late (L) and very late (VL) replication timing contexts in cancer types other than those shown in Figure 3. C) Boxplot showing distributions of weight of Signature SBS8 in very low (VL), low (L), high (H) and very high (VH) replication speed contexts in cancer types other than those shown in Figure 3.

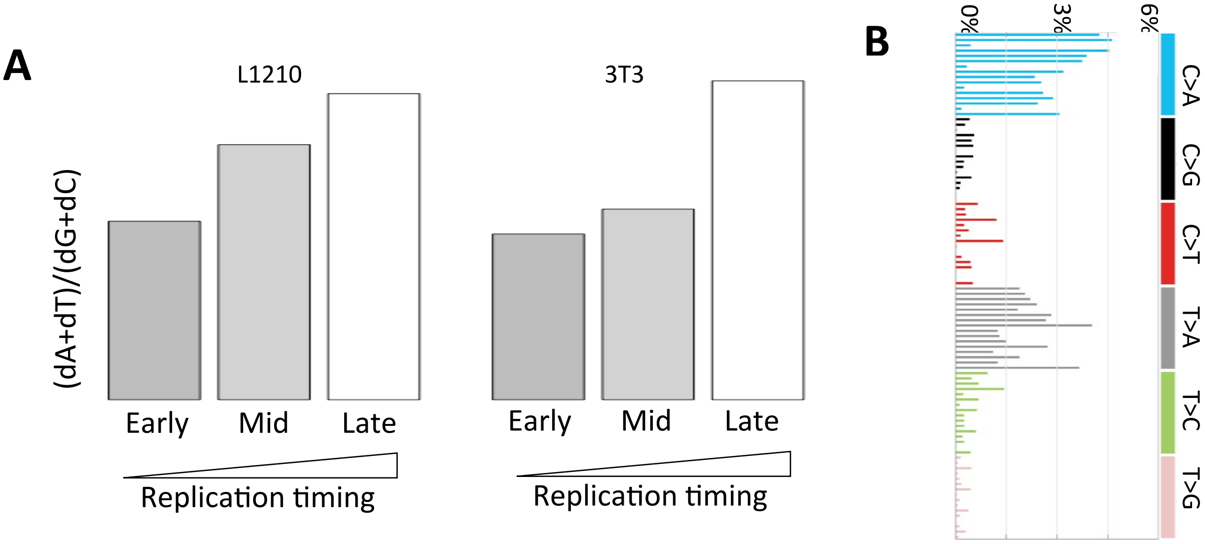

Supplementary Figure 4: A) Schematic representation of imbalance of dNTP pools during the progression of replication results in proportional increase of dATP+dTTP relative to dGTP and dCTP in L1210 and 3T3 cell lines (Kenigsberg et al. Nucleic Acids Res, 2016). B) Relative abundance of C>A:G>T and T>A:A>T substitutions in SBS8 mutational signatures.

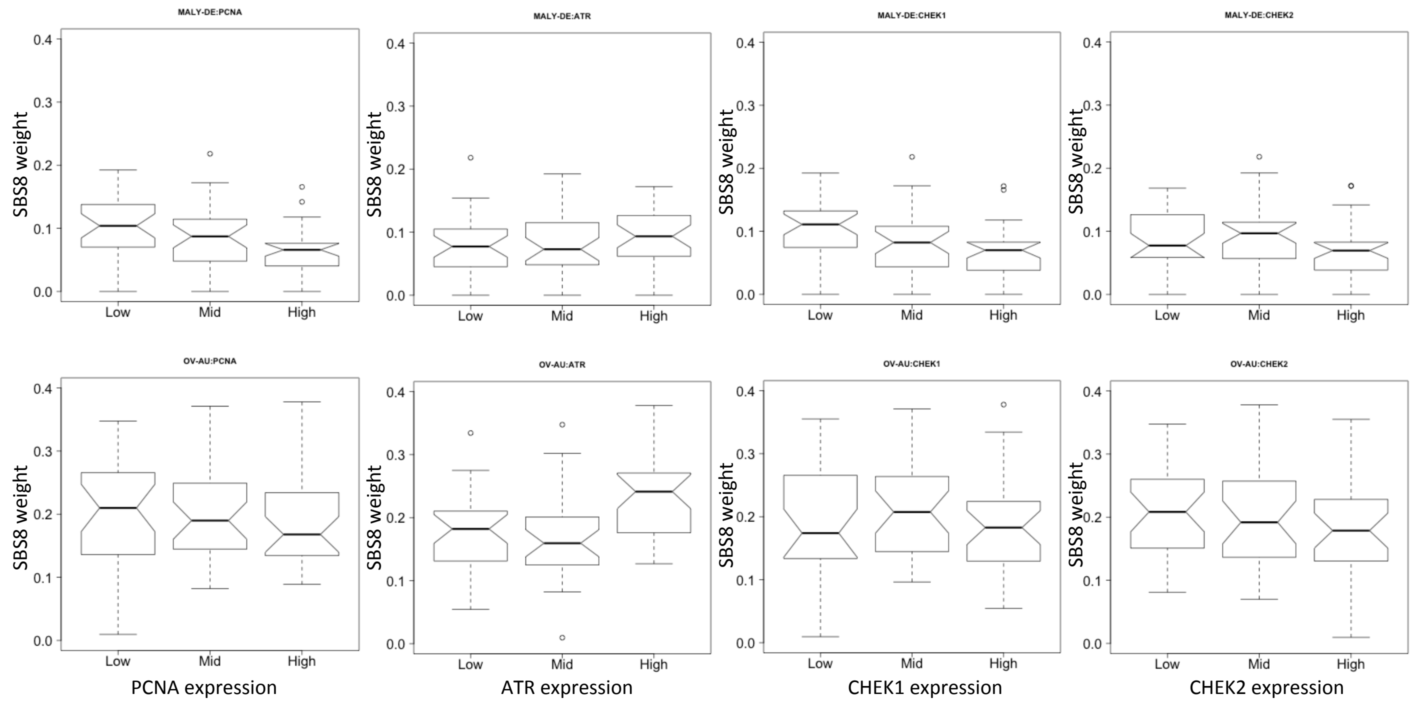

Supplementary Figure 5: Boxplot showing proportion of SBS8 in late replicating regions in the tumors from different cancer cohorts, grouped according to tumor purity-adjusted low, medium, and high expression of replication related gene PCNA, DNA damage sensing ATR, and cell cycle checkpoint genes CHEK1 and CHEK2.

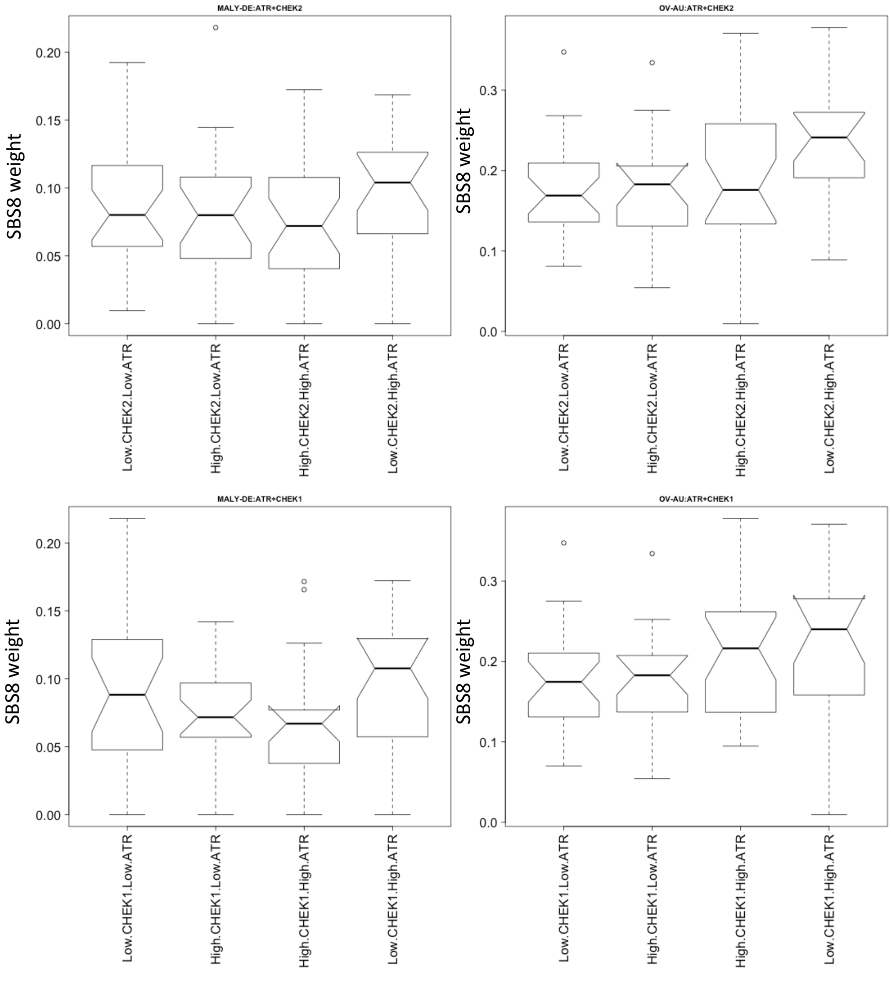

Supplementary Figure 6: Boxplot showing proportion of SBS8 in late replicating regions in the tumors from lymphoma and ovarian cancer cohorts, grouped according to tumor purity-adjusted low, medium, and high expression of ATR and CHEK2.

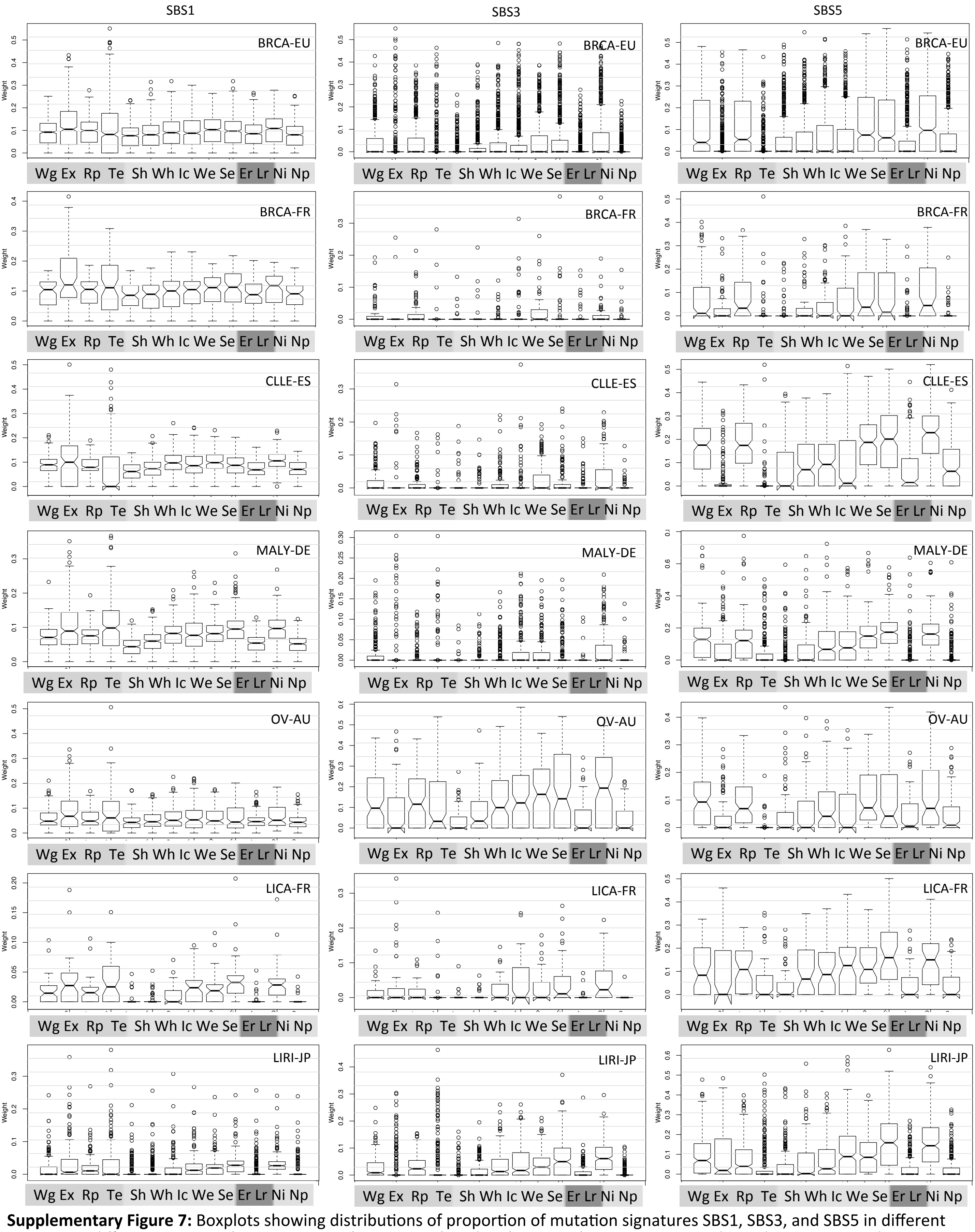

Supplementary Figure 7: Boxplots showing distributions of proportion of mutation signatures SBS1, SBS3, and SBS5 in different contexts in different cancer cohorts. Cancer cohort names listed in the top right corner are as indicated in Figure 1 and Supplementary Table 1. X-axis labels are: Wg: whole gene, Ex: exon, Rp: repeat, Te: telomere, Sh: strong heterochromatin, Wh: weak heterochromatin, Ic: intermediate chromatin, We: weak euchromatin, Se: strong euchromatin Er: early replicating regions, Lr: late replicating regions, Ni: nuclear interior, Np: nuclear periphery.

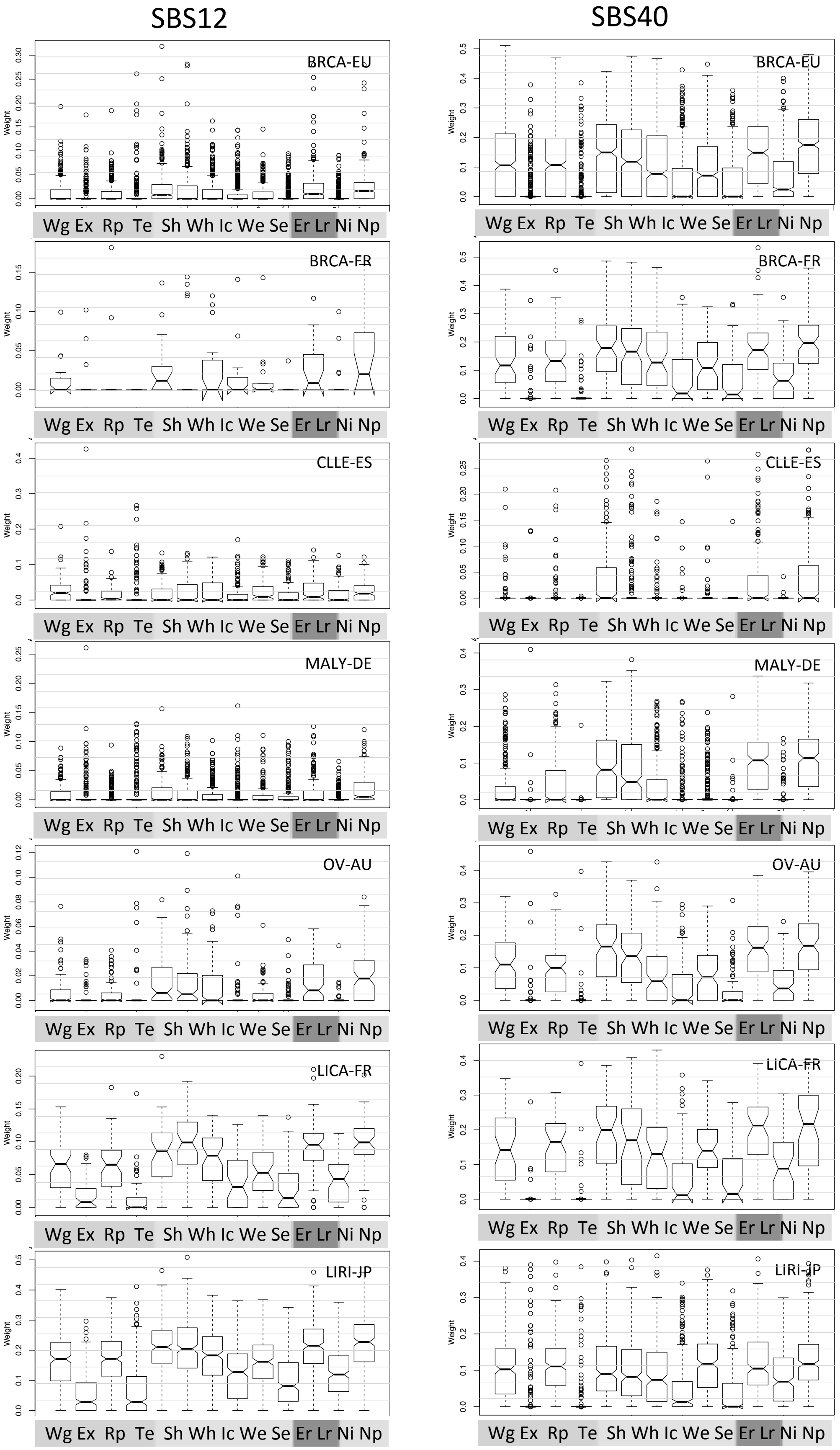

Supplementary Figure 8: Boxplots showing distributions of proportion of mutational signatures SBS12 and SBS40 in different contexts in selected cancer cohorts. Cancer cohort names listed in the top right corner are as indicated in Figure 1 and Supplementary Table 1. X-axis labels are: Wg: whole gene, Ex: exon, Rp: repeat, Te: telomere, Sh: strong heterochromatin, Wh: weak heterochromatin, Ic: intermediate chromatin, We: weak euchromatin, Se: strong euchromatin Er: early replicating regions, Lr: late replicating regions, Ni: nuclear interior, Np: nuclear periphery.

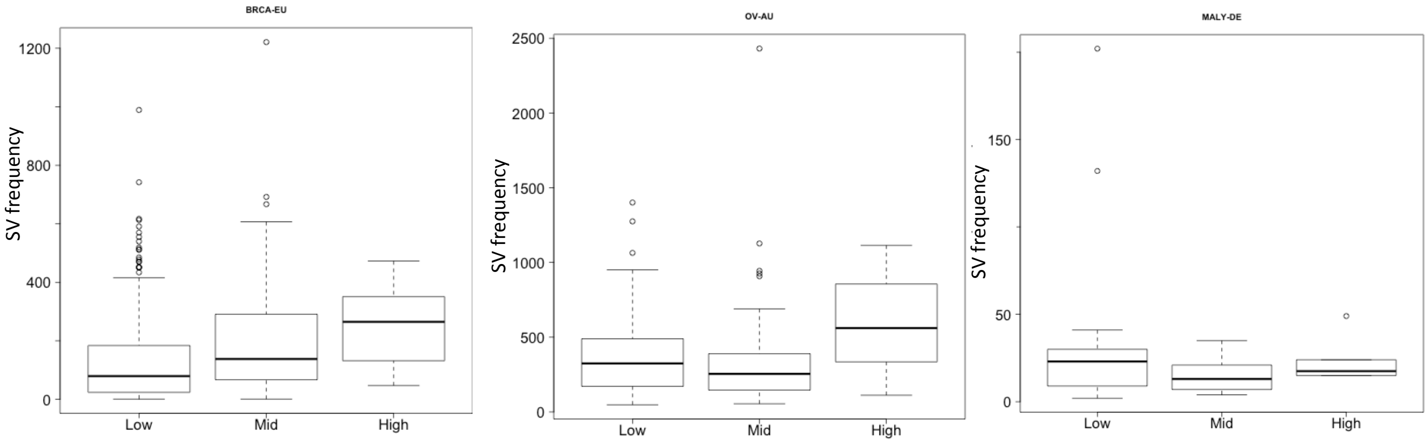

Supplementary Figure 9: Boxplot showing frequency of somatic structural variations in tumors grouped according to low, mid, and high proportion of SBS8 genome-wide (0-33, 33-67, 67-100 percentile within respective cancer types) in the tumors from different cancer cohorts. Similar results were obtained when the samples were grouped according to proportion of SBS8 only in late replicating regions. Consistent results were obtained for other cohorts, including those representing similar cancer types (e.g. BRCA-FR).

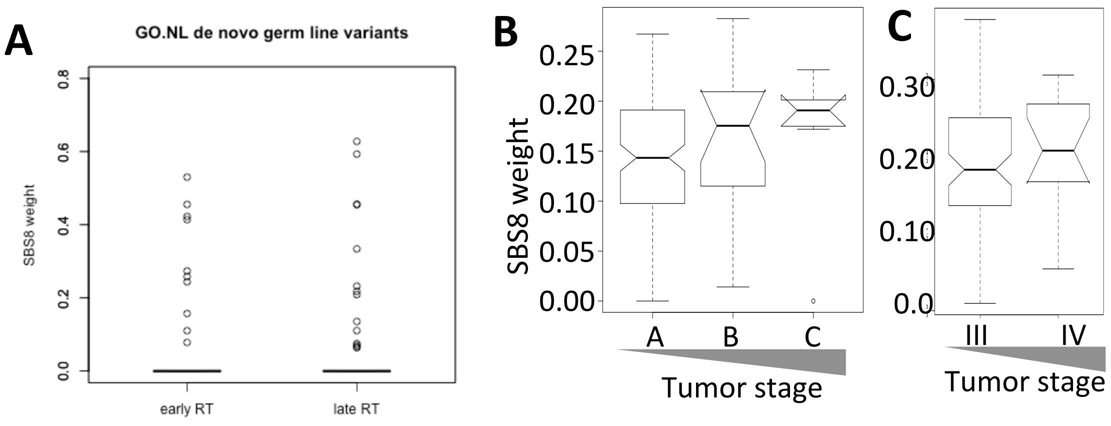

Supplementary Figure 10: A) Analyzing 11,020 de novo germ line mutations in 250 families from the Netherlands Genome project, proportions of SBS8 in tissue invariant early and late replicating regions are shown using boxplots. SBS8 weight increases with tumor stage in B) chronic lymphocytic leukemia and C) ovarian cancer.
