# Supplementary material for: Mutational signature SBS8 predominantly arises due to late replication errors in cancer
Source: Commun Biol. 2020 Aug 3;3:421. doi: 10.1038/s42003-020-01119-5 (PMC7400754; doi:10.1038/s42003-020-01119-5)
Supplement: Supplementary file 7 — Reporting Summary [file 42003_2020_1119_MOESM7_ESM.pdf]

Corresponding author(s): Subhajyoti De

Last updated by author(s): Jun 7, 2020

Reporting Summary

Nature Research wishes to improve the reproducibility of the work that we publish. This form provides structure for consistency and transparency in reporting. For further information on Nature Research policies, see our Editorial Policies and the Editorial Policy Checklist.

Statistics

For all statistical analyses, confirm that the following items are present in the figure legend, table legend, main text, or Methods section.

n/a	Confirmed	
☐	☒	The exact sample size (*n*) for each experimental group/condition, given as a discrete number and unit of measurement
☐	☒	A statement on whether measurements were taken from distinct samples or whether the same sample was measured repeatedly
☐	☒	The statistical test(s) used AND whether they are one- or two-sided<br>*Only common tests should be described solely by name; describe more complex techniques in the Methods section.*
☐	☒	A description of all covariates tested
☐	☒	A description of any assumptions or corrections, such as tests of normality and adjustment for multiple comparisons
☐	☒	A full description of the statistical parameters including central tendency (e.g. means) or other basic estimates (e.g. regression coefficient) AND variation (e.g. standard deviation) or associated estimates of uncertainty (e.g. confidence intervals)
☐	☒	For null hypothesis testing, the test statistic (e.g. *F*, *t*, *r*) with confidence intervals, effect sizes, degrees of freedom and *P* value noted<br>*Give P values as exact values whenever suitable.*
☒	☐	For Bayesian analysis, information on the choice of priors and Markov chain Monte Carlo settings
☒	☐	For hierarchical and complex designs, identification of the appropriate level for tests and full reporting of outcomes
☒	☐	Estimates of effect sizes (e.g. Cohen's *d*, Pearson's *r*), indicating how they were calculated

Our web collection on statistics for biologists contains articles on many of the points above.

Software and code

Policy information about availability of computer code

Data collection	1. Gene expression data and WGS somatic mutation data for cancer cohorts was obtained from ICGC data portal release 28.<br>2. Cell line/ tissue specific Replication timing data was obtained from 'replication domain database' (https://www2.replicationdomain.com/database.php)<br>3. Tissue invariant Epi/Genomic context data was take from the publication 'Nuclear Topology Modulates the Mutational Landscapes of Cancer Genomes' (https://www.nature.com/articles/nsmb.3474)<br>4. Consensus single base substitution (SBS) mutational signatures (version 3) from the COSMIC database (https://cancer.sanger.ac.uk/cosmic/signatures)
Data analysis	1. The context of somatic mutations was obtained by an r-package i.e., MutSigTools , developed by our group. (https://github.com/sjdlabgroup/MutSigTools). 'MutsigTools' use 'deconstructSigs' package for extraction of mutational signatures from the mutational catalogue of cancer cohorts (https://github.com/raerose01/deconstructSigs)<br>2. De-Novo extraction mutational signatures were obtained by 'SigProfiler' (https://github.com/AlexandrovLab/SigProfilerExtractorR)

For manuscripts utilizing custom algorithms or software that are central to the research but not yet described in published literature, software must be made available to editors and reviewers. We strongly encourage code deposition in a community repository (e.g. GitHub). See the Nature Research guidelines for submitting code & software for further information.

Data

Policy information about availability of data

All manuscripts must include a data availability statement. This statement should provide the following information, where applicable:

- Accession codes, unique identifiers, or web links for publicly available datasets
- A list of figures that have associated raw data
- A description of any restrictions on data availability

This study used publicly available datasets. Composite epigenomic states are provided as Supplementary data. Any other data are available from the corresponding authors upon request.

Field-specific reporting

Please select the one below that is the best fit for your research. If you are not sure, read the appropriate sections before making your selection.

☒ Life sciences ☐ Behavioural & social sciences ☐ Ecological, evolutionary & environmental sciences

For a reference copy of the document with all sections, see nature.com/documents/nr-reporting-summary-flat.pdf

Life sciences study design

All studies must disclose on these points even when the disclosure is negative.

Sample size	We analyzed somatic point mutation data for multiple cancer types from the International Cancer Genome Consortium (ICGC v28)13. After removing samples with <500 somatic point mutations from whole genome sequencing, we had 20-569 samples (median: 145) per cohort for downstream analyses. A summary of the cohorts included in the study are listed in Figure 1B and Supplementary Table 1.
Data exclusions	Our key conclusions did not change after excluding the ENCODE back-listed genomic regions that are prone to technical artefacts (http://hgdownload.cse.ucsc.edu/goldenpath/hg19/encodeDCC/wgEncodeMapability/ wgEncodeDacMapabilityConsensusExcludable.bed.gz).
Replication	Analysis was performed on multiple cancer cohorts, a subset of which represented similar cancer types (e.g. BRCA-EU, BRCA-UK and BRCA-FR), and key conclusions were consistent.
Randomization	Not applicable
Blinding	Not applicable

Reporting for specific materials, systems and methods

We require information from authors about some types of materials, experimental systems and methods used in many studies. Here, indicate whether each material, system or method listed is relevant to your study. If you are not sure if a list item applies to your research, read the appropriate section before selecting a response.

Materials & experimental systems

n/a	Involved in the study	
☒	☐	Antibodies
☒	☐	Eukaryotic cell lines
☒	☐	Palaeontology and archaeology
☒	☐	Animals and other organisms
☒	☐	Human research participants
☒	☐	Clinical data
☒	☐	Dual use research of concern

Methods

n/a	Involved in the study	
☒	☐	ChIP-seq
☒	☐	Flow cytometry
☒	☐	MRI-based neuroimaging
