# Supplementary material for: Mutational signature SBS8 predominantly arises due to late replication errors in cancer
Source: Commun Biol. 2020 Aug 3;3:421. doi: 10.1038/s42003-020-01119-5 (PMC7400754; doi:10.1038/s42003-020-01119-5)
Supplement: Supplementary file 8 — Description of Additional Supplementary Files [file 42003_2020_1119_MOESM8_ESM.pdf]

DESCRIPTION OF SUPPLEMENTARY DATA FILES

Supplementary Data 1: Summary of the samples from different cancer types
Supplementary Data 2: Interpretation of MRE states from a 20 state model
Supplementary Data 3: Genome-wide annotation of MRE states in different cell types
Supplementary Data 4: Weight of mutational signature 8 in tssue invariant early and late replication timing regions in the cancer samples
Supplementary Data 5: Mutation signatures whose proportions in early replicating regions correlate with the proportions of SBS8 in late replicating regions (p-value <0.05, spearman correlation value >0.2 or < -0.2 are shown
